# Delayed Antibiotic Prescription by General Practitioners in the UK: A Stated-Choice Study

**DOI:** 10.3390/antibiotics9090608

**Published:** 2020-09-16

**Authors:** Liz Morrell, James Buchanan, Laurence S. J. Roope, Koen B. Pouwels, Christopher C. Butler, Benedict Hayhoe, Michael V. Moore, Sarah Tonkin-Crine, Monsey McLeod, Julie V. Robotham, A. Sarah Walker, Sarah Wordsworth, on behalf of the STEPUP team

**Affiliations:** 1 Health Economics Research Centre, Nuffield Department of Population Health, University of Oxford, Oxford OX3 7LF, UK; james.buchanan@dph.ox.ac.uk (J.B.); laurence.roope@dph.ox.ac.uk (L.S.J.R.); koen.pouwels@ndph.ox.ac.uk (K.B.P.); sarah.wordsworth@dph.ox.ac.uk (S.W.); 2 NIHR Health Protection Research Unit in Healthcare Associated Infections and Antimicrobial Resistance, University of Oxford, Oxford OX2 6GG, UK; christopher.butler@phc.ox.ac.uk (C.C.B.); sarah.tonkin-crine@phc.ox.ac.uk (S.T.-C.); sarah.walker@ndm.ox.ac.uk (A.S.W.); 3 NIHR Biomedical Research Centre Oxford, John Radcliffe Hospital, University of Oxford, Oxford OX3 9DU, UK; 4 Nuffield Department of Primary Care Health Sciences, University of Oxford, Oxford OX2 6GG, UK; 5 Department of Primary Care and Public Health, School of Public Health, Imperial College London, London W2 1PG, UK; b.hayhoe@imperial.ac.uk; 6 Primary Care, Population Sciences and Medical Education, Faculty of Medicine, University of Southampton, Southampton SO17 1BJ, UK; mvm198@soton.ac.uk; 7 NIHR Health Protection Research Unit in Healthcare-Associated Infections and Antimicrobial Resistance, Imperial College London, London SW7 2AZ, UK; monsey.mcleod@nhs.net; 8 Centre for Medication Safety and Service Quality, Pharmacy Department, Imperial College Healthcare NHS Trust, London W2 1NY, UK; 9 NIHR Imperial Patient Safety Translational Research Centre, Imperial College London, London SW7 2AZ, UK; 10 Modelling and Economics Unit, National Infection Service, Public Health England, London SE1 8UG, UK; julie.robotham@phe.gov.uk; 11 Nuffield Department of Medicine, University of Oxford, Oxford OX3 7BN, UK

**Keywords:** antibiotic resistance, choice experiment, primary care, general practice, delayed prescription, respiratory tract infection, stewardship, UK

## Abstract

Delayed antibiotic prescription in primary care has been shown to reduce antibiotic consumption, without increasing risk of complications, yet is not widely used in the UK. We sought to quantify the relative importance of factors affecting the decision to give a delayed prescription, using a stated-choice survey among UK general practitioners. Respondents were asked whether they would provide a delayed or immediate prescription in fifteen hypothetical consultations, described by eight attributes. They were also asked if they would prefer not to prescribe antibiotics. The most important determinants of choice between immediate and delayed prescription were symptoms, duration of illness, and the presence of multiple comorbidities. Respondents were more likely to choose a delayed prescription if the patient preferred not to have antibiotics, but consultation length had little effect. When given the option, respondents chose not to prescribe antibiotics in 51% of cases, with delayed prescription chosen in 21%. Clinical features remained important. Patient preference did not affect the decision to give no antibiotics. We suggest that broader dissemination of the clinical evidence supporting use of delayed prescription for specific presentations may help increase appropriate use. Establishing patient preferences regarding antibiotics may help to overcome concerns about patient acceptance. Increasing consultation length appears unlikely to affect the use of delayed prescription.

## 1. Introduction

Reducing unnecessary antibiotic consumption is essential to reduce selection pressure on bacteria to develop resistance, and preserve the effectiveness of existing antibiotics [1,2]. In the UK, over 70% of antibiotics are prescribed in primary care [3], with 32 million antibiotic prescriptions dispensed in England in 2019 [4]. Much of this prescribing may be avoidable [5]. Many conditions treated in primary care, such as a substantial proportion of respiratory tract infections, are self-limiting and will resolve without antibiotics [5,6].

Amongst the strategies aimed at reducing antibiotic consumption, one option in primary care is delayed (or ‘back-up’) prescriptions. With this approach, a prescription is issued, but the patient is advised to wait, and only collect and begin taking the antibiotics if their symptoms worsen or do not resolve within a specified time period. This strategy can be used where the prescriber believes that antibiotics are not needed at the time, but has some clinical uncertainty as to whether the condition could deteriorate without antibiotics [7]. The delayed prescription approach has been shown in randomised trials in primary care to be effective in reducing consumption with little effect on symptom control or risk of complications, in respiratory tract infections [8,9,10,11], conjunctivitis [12] and urinary tract infections [13]. In the UK, the National Institute for Health and Care Excellence (NICE) has included the use of delayed prescriptions in its guidelines and Clinical Knowledge Summaries since 2008 [14,15,16,17,18]. The approach has the potential to provide reassurance to both prescribers [19] and patients [20]; it provides easier access to antibiotics should they be needed, but unnecessary consumption may be avoided if the illness follows its expected course. However, despite the supporting evidence, use of delayed prescriptions has been limited, with studies showing that only around 14% of prescriptions for common infections are delayed prescriptions [21,22]. To support more widespread implementation of this strategy for antimicrobial stewardship (in line with NICE guidelines), it is important to understand the barriers to using delayed prescriptions.

Studies to date on primary care physicians’ attitudes to delayed prescription have been predominantly qualitative. These studies highlighted concerns over delayed prescriptions giving a potentially ambiguous message to patients, abdication of clinical responsibility and time taken in the consultation to explain a delayed prescription to the patient [19,23,24,25,26]. Our study aimed to develop this evidence base further by providing quantitative information on the relative importance of factors in the decision to use a delayed prescription as an alternative to an immediate prescription, with a focus on factors relating to the patient and the information discussed during the consultation.

Our study setting is primary care in the UK’s National Health Service (NHS), where physicians (known as general practitioners (GPs)) make the majority of antibiotic prescribing decisions. We focus on respiratory tract infections (RTI), which are among the most common reasons for GP consultations [14,27], and account for a high proportion of antibiotic prescribing in primary care [28,29]. A recent analysis indicated that in the years 2013–2015, at least 32% of primary care antibiotic prescriptions were for RTIs (including ear, nose and throat conditions) [28]; this represents over 11 million prescriptions in England at the 2018 prescribing level of 625 antibiotic prescriptions per 1000 patients [3]. In particular, we focus on cough and sore throat symptoms, for which much of the antibiotic prescribing is likely to be unnecessary [5,30,31]. There is therefore significant potential to reduce prescribing safely in this condition via broader use of delayed prescriptions where clinically appropriate.

We conducted a stated-choice study, a survey method widely used in health research [32,33,34]. The method asks respondents to make choices between alternative healthcare options, which are designed to require trade-offs between the attributes of these options. Our study presented a sample of UK GPs with fifteen hypothetical consulting scenarios they might encounter when a patient presents with an RTI, and asked them to choose whether they would give the patient an immediate or delayed prescription. In each case, respondents were also asked if they would prefer to give no antibiotic prescription. The scenarios consisted of eight attributes that described the presenting condition, the patient and the consultation (Table 1). Respondent choices were analysed using logistic regression, to determine the relative influence of the attributes on the prescribing decision.

## 2. Results

### 2.1. Respondent Characteristics

A total of 181 GPs completed the survey, with a median completion time of 17 min. The sample was consistent with the target distributions for sex, age, country and practice size, reflecting the population of UK GPs (Table 2). One in five respondents reported finding the survey difficult to complete to some degree.

Half of the respondents were partners in their GP practice. The majority considered the practice where they work most often to be of medium level of deprivation, and average level of antibiotic prescribing. On average, respondents reported that 17% of their patients who present with an RTI leave with a delayed antibiotic prescription, but there was wide variation in prescribing patterns between individuals; eight respondents reported never using delayed antibiotic prescriptions for patients with RTIs.

### 2.2. Importance Ranking of the Attributes

**Figure 1:**
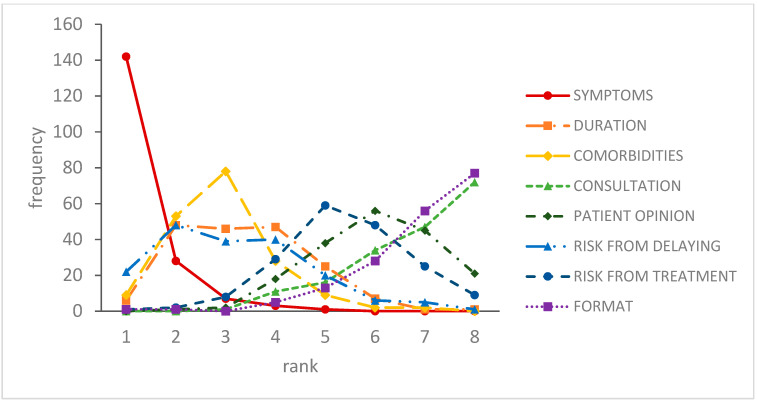
Ranking of attribute importance. Attribute descriptions: DURATION: duration of illness prior to consultation; CONSULTATION: length of consultation; PATIENT OPINION: preferences regarding antibiotics expressed by the patient; RISK FROM DELAYING: risk of harm from not starting antibiotics straight away; RISK FROM TREATMENT: risk of adverse effects from taking antibiotics; FORMAT: how the delayed prescription would be provided. Vertical axis indicates the number of respondents who ranked a given attribute at the rank shown on the horizontal axis (1 = highest rank).

Figure 1 shows the number of respondents who assigned each attribute a given rank, before completing the choice questions. Symptoms were the most important, followed by comorbidities, duration of illness and the risk of harm due to delaying treatment. Respondents ranked length of consultation, the format of the delayed prescription and patient preference as the least important.

### 2.3. Choice Responses

**Figure 2:**
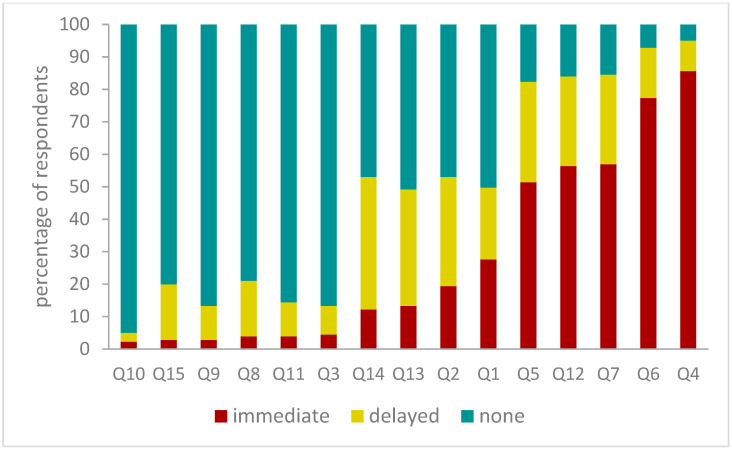
Proportions ultimately choosing immediate, delayed and no prescription per choice question. Q: choice question, numbered by the order in which they were presented to respondents. To illustrate the patterns in proportions choosing immediate, delayed, or no prescription, the graph orders the bars by the proportion ultimately choosing the immediate prescription.

Each of the 181 GPs answered 15 choice questions, resulting in 2715 choice occasions. Initially, 68% of choices between delayed and immediate prescription were for a delayed prescription. When the no-prescription alternative was offered, 51% (1393/2715) of choices were for this option, with the vast majority of those choices switching from an initial choice of delayed prescription (1383 of the 1393 no-prescription choices). This left 21% and 28% remaining with their original choice of delayed or immediate prescription respectively. Twenty-six respondents never chose a delayed prescription, with fewer never choosing immediate or no prescription (five and two respondents respectively), and 95% of respondents choosing a delayed prescription six times or fewer. By question, the proportions choosing immediate and no prescription were inversely correlated. The trend in proportion choosing delayed prescription was less clear, but appeared to be higher in the scenarios where there was no strong preference for either immediate or no prescription (Figure 2).

### 2.4. Choice Modelling

Table 3 presents a mixed-effect logistic regression model, which estimates the effect of each of the scenario attributes on the likelihood of respondents choosing the delayed prescription option over an immediate prescription. By using a mixed-effect logistic regression, the model allows for differences between respondents in their tendency to choose the delayed prescription. The coefficients are all of the expected sign, giving the model face validity; that is, positive where we would expect an increase in the attribute to increase the likelihood of respondents choosing the delayed prescription, and negative where we would expect the likelihood to decrease. The attributes in the model explain 61% of the variation in responses; this rises to 65% when between-respondent heterogeneity is incorporated.

Respondents were more likely to choose the delayed prescription for the minor versions of both upper and lower respiratory tract symptoms. The probability of choosing the delayed prescription increased by 0.41 for productive cough and runny nose compared to the more serious lower tract symptoms, and by 0.54 for the minor throat symptoms compared to the more serious (for full table of the effects on the marginal probability of choosing delayed prescription including confidence intervals, see Supplementary Material S1 Section 2). Respondents were also more likely to choose the delayed prescription if the consultation was longer, or there was a higher risk of adverse events from taking antibiotics, but these effects were small (probability of choosing the delayed prescription increased by 0.005 per additional minute of consultation or 0.003 per 1% increase in risk of adverse effects). They were less likely to choose delayed prescription if the symptoms had been present for longer (probability decreased by 0.03 per day), if the patient had multiple comorbidities (probability decreased by 0.12 compared to no comorbidities), and with increasing risk of harm from delaying treatment (probability decreased by 0.01 per 1% increased risk). A delayed prescription was more likely if the patient expressed a preference not to take antibiotics (probability increased by 0.03), and conversely, less likely if the patient preferred to have antibiotics (probability decreased by 0.04). Compared to the most common format of delayed prescription (giving the patient a prescription with advice to wait), respondents were less likely to choose delayed prescription if the patient would have to return to the surgery to collect it (decrease in probability of choosing the delayed prescription of 0.04).

The relative strength of the attribute effects can be seen by comparing the magnitude of the coefficients. A patient expressing a preference to have antibiotics, for example, had a similar effect to an additional day of illness in reducing the probability of the GP choosing a delayed prescription. Similarly, a patient with a serious chest infection of a given duration, and a patient who had had a minor infection for 10 days longer, would have a similar probability of receiving a delayed prescription. The effect of a 1% difference in the risk from delaying antibiotics was approximately four-fold greater than the effect of a 1% difference in risks due to antibiotic treatment.

When respondent characteristics were added (Table 3), allowing for self-reported prescribing behaviour improved the fit of the model; respondents who reported a high level of immediate prescribing for RTIs in practice were more likely to choose the immediate prescription in the study. Although the effect per 1% difference in self-reported immediate prescribing was modest, the proportion of self-reported immediate prescriptions for RTI ranged from 1% to 90%; at the mean value of 31% immediate prescriptions in practice, the effect was equivalent to 2.3 additional days of illness in reducing the likelihood of a delayed prescription. Other respondent characteristics showed no evidence of an effect on respondents’ choices.

The model was robust to the exclusion of respondents who chose delayed prescription for the practice question (*n* = 27), chose the same response to all questions (*n* = 6), completed the survey in the fastest 1–5 percentiles (*n* = 9) or who reported that they found the survey ‘quite difficult’, ‘difficult’ or ‘very difficult’ (*n* = 39). The coefficients varied slightly, but the conclusions were unchanged.

**Figure 3:**
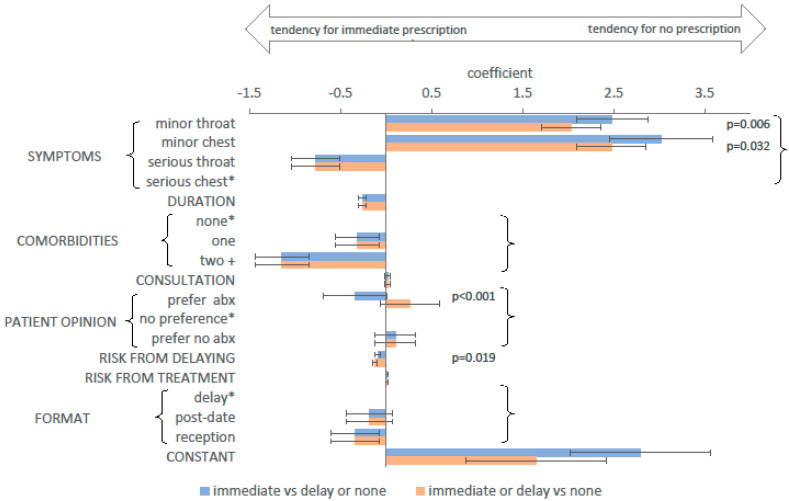
Coefficients for the ordered logistic regression model. Symptoms descriptions: minor throat—sore throat and swollen glands; minor chest—chesty cough and runny nose; serious throat—sore throat, swollen glands and fever; serious chest—chesty cough, fever and pain on breathing. * Reference level for categorical variables. p-values are shown where the coefficients differed (p < 0.05) between the choice to give an immediate prescription, and the choice to prescribe at all. For all other attributes and levels, the p-value for this difference was greater than 0.05, and the coefficients were constrained to be equal in the model. Bars indicate 95% confidence intervals. Abbreviations: abx—antibiotics.

An ordered logistic regression model allows for the additional choice of ‘no prescription’ (Figure 3). This assumes the three possible outcomes have a natural order (immediate, delayed, and no prescription) and models the probability of respondents choosing each outcome relative to the adjacent one in the hierarchy. Where the two coefficients for a given attribute are not significantly different (*p* > 0.05, Wald test), those coefficients are assumed by the model to be equal (equal bars in Figure 3). Positive coefficients indicate that the attribute increased the likelihood of a reduced prescribing choice (no or delayed prescription), and negative coefficients indicate an increase in the likelihood of any prescription.

The direction and relative size of the effect of the attributes was similar to those described above (see Supplementary Material S1 Section 3 for a direct comparison). For most attributes, there was no difference between their effect on the decision between an immediate prescription and a reduced prescribing choice, and their effect on the decision to prescribe (delayed or immediate) or not. However, four of the coefficients suggested the attribute had a different effect depending on the type of decision being made. A patient expressing a preference to have antibiotics may influence the type of prescription, but there was no evidence that patient preferences affected the choice to prescribe at all. Similarly, minor symptoms had a stronger effect on the decision not to give an immediate prescription than on whether to prescribe at all. There was a minor difference in the effect of the risk of delaying treatment.

**Figure 4:**
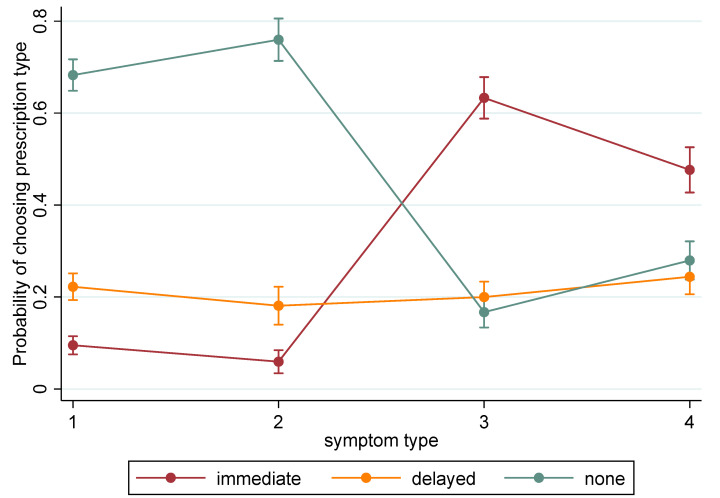
Probability of choosing immediate, delayed or no prescription, for each symptom type. Symptom descriptions: 1 minor throat—sore throat and swollen glands; 2 minor chest—chesty cough and runny nose; 3 serious throat—sore throat, swollen glands and fever; 4 serious chest—chesty cough, fever and pain on breathing. Bars indicate 95% confidence intervals.

Figure 4 shows the probability of choosing each prescription type for each of the four types of symptoms presented in the survey, as predicted by the ordered logistic regression model. No prescription was the most likely choice for the minor symptoms, whilst an immediate prescription was most likely for the more serious symptoms. The proportion of delayed prescriptions remained almost constant across the four symptom levels. A similar pattern was seen for the other categorical attributes (see Supplementary Material S1 Section 4).

## 3. Discussion

Our study suggests that clinical indicators—presenting symptoms, duration of illness and patient comorbidities—are important factors in the decision to use delayed prescription. Patient preferences have some influence on the decision between immediate and delayed prescription, and GPs are less likely to use delayed prescription if the patient would have to come back to the practice to collect the prescription. The risk from delaying antibiotic treatment has a greater effect on the decision than the risk due to antibiotic treatment, demonstrating greater risk adversity regarding adverse outcomes than side effects. There is little effect of consultation length on prescription choice.

To our knowledge this is the first large-scale study to quantify the trade-offs made by UK GPs in considering delayed prescription. A smaller (*n* = 23) choice study of prescribing decisions among Australian GPs examined factors affecting antibiotic prescribing overall. Two attributes shared with our study (patient preferences and duration of illness) had significant effects in both studies; the effect of patient preferences was less in our study, possibly because of the strong effect of symptoms in our study, which were held constant in the Australian work [26]. Our findings are consistent with qualitative observations on the importance of perceived patient expectations in different countries [19,24,25,26,43,44]. These similarities suggest our results may be generalisable to other settings with similar primary healthcare systems.

The relative importance of the attributes from the regression models is broadly consistent with respondents’ importance rankings. Both indicate that concerns about the potential for harm due to delaying antibiotics is more important in the prescribing decision than the risk of adverse effects due to taking antibiotics. This may reflect the doctor’s role to treat patients, so the risk of a negative outcome from ‘doing nothing’ may be seen as worse than the risk of a negative outcome from giving treatment. Alternatively, it may be an acknowledgement that in a situation where antibiotics are indicated, the benefit from prescribing is accepted to outweigh the risk of harm from doing so. The length of the consultation was ranked low, consistent with its modest effect in the choice questions, despite concerns raised in qualitative studies over the time taken to explain the delayed prescription [19]. Our findings suggest that increasing the length of consultations to allow for the explanation required would not necessarily increase the uptake of delayed prescription.

Given the similarity of the coefficients in the two types of model, and the observation that the majority of ‘no prescription’ choices were originally for delayed prescription, we have not found a unique model for delayed prescription. Rather, our results appear to reflect a spectrum from immediate to no need for antibiotics, and our models indicate factors that move a GP’s prescribing decision towards reduced prescribing—that is, to delayed or no prescription. This is perhaps consistent with findings in observational studies of delayed prescription, that the population of patients given a delayed prescription show a symptom distribution that is intermediate between those offered immediate or no prescription [10,21]. It may be that this rather loosely defined intermediate position contributes to the uncertainty in using delayed prescription noted by some GPs in qualitative work [19,26], thus reducing its use.

The serious sore throat symptoms (sore throat, swollen glands and fever) had a greater negative effect on the likelihood of GPs using delayed or no prescription than serious chest symptoms (chesty cough, fever and pain on breathing). This is perhaps counter-intuitive given the potential seriousness of bacterial pneumonia, and may be due to variation in the way respondents interpreted the descriptions, for example assuming pneumonia had been excluded, or that the pain in breathing was due to coughing. Alternatively, respondents with recent experience with streptococcal pharyngitis or rising incidence of scarlet fever [45,46], may have been sensitised to be more concerned about throat symptoms.

Based on our results, we suggest two possible approaches for continued reduction of antibiotic prescribing. First, our findings concur with other work recommending support for GPs in handling perceived patient pressure to prescribe [23,26,47]. In addition, we recommend patients are encouraged to express any preference not to have antibiotics (or a neutral opinion), and GPs to ask questions to establish that preference during the consultation. Although patients preferring not to have antibiotics was not consistently a strong factor in determining the prescribing decision in our models, we note that GPs’ perceptions of patients’ preferences are not necessarily accurate [48,49,50,51]. Hearing the patient’s actual preference would avoid assumption that the patient expects antibiotics; this would also reduce concerns about delayed prescription giving an ambiguous message to the patient if it is clearly in line with the patient’s preference.

Second, whilst symptoms and their duration are a key driver of prescribing behaviour, some GPs appear to be more averse to delayed or no antibiotic prescribing than would be expected from clinical evidence and guidelines. Reasons for this variation could include an individual’s previous negative experience, tolerance of risk or ambiguity, or the mechanisms available for keeping up to date with data and guidelines. Raising awareness and understanding of the trial data supporting the use of delayed prescription may be helpful in reducing prescribing overall, including use of delayed prescription. A ‘one-stop’ website containing the key evidence for specific presentations, and current guidance, along with peer suggestions for explaining prescribing decisions to patients, may be helpful.

Some practitioners oppose the use of delayed prescription [52]. Two subgroups identified in our study could reflect views of this type: respondents who never chose the delayed prescription option in the study, and respondents who reported never using delayed antibiotic prescription in practice. There was minimal overlap between these two groups, and the groups were too small to draw any conclusions from our data. Further work to understand these polarised opinions would be helpful—particularly if they tend to be high prescribers—to identify alternative routes to support them in reducing antibiotic use.

### Limitations

The study is limited by its hypothetical nature, meaning the responses may be idealised and not reflect actual practice. We suggest this may be particularly true of studies such as this where experts respond in their professional capacity, in contrast to general population or patient studies where we are seeking opinions; here, there may be consistency stemming from professional training or best practice guidance, and respondents might wish to reflect that in their responses. This may explain the relatively low heterogeneity of responses seen in this study. However, concerns that responses do not reflect actual practice are somewhat allayed by the observation that reported prescribing behaviour in practice not only varied widely, but was a significant predictor of responses to the choice questions.

Further, in constructing the scenarios, the number of attributes was inevitably constrained in order to manage respondent burden, and the scenarios may have omitted important features (for example, additional information about the patient, social factors, or clinical findings) that would affect the results. Our study included eight attributes; this is relatively high compared to studies among members of the public, which mostly use six or fewer [32,33,53], and a fifth of respondents reported finding the choices difficult. However, GPs are used to making complex decisions in their clinical practice; further, free-text comments on the survey did not raise complexity as an issue, and the relatively low heterogeneity of responses suggests that even if the choices were difficult, respondents were able to make consistent choices. Nonetheless, respondents may have used simplifying heuristics, such as ignoring certain attributes; for example, it is possible that respondents assessed the risk of recurrence or progression from the clinical features, and paid reduced attention to the attribute that quantified that risk.

Our choice question was presented in two parts, with the second allowing the option of no antibiotic prescription. This was to allow us to capture data on the choice between immediate and delayed prescription, our primary interest. However, it may not reflect the actual decision process in practice, which may have introduced bias to the choices. It may be that, having made a decision, respondents were more reluctant to change their mind and switch to ‘no prescription’, so overestimating the likelihood of prescribing an antibiotic and reducing the estimated impact of the attributes. However, the results show that 51% of choices switched in this way, suggesting it did not have a major impact.

The study design was optimised to quantify the main effects of the attributes, but did not allow for evaluation of interactions between the attributes. Some exploratory analyses were attempted, but the findings were difficult to interpret because of collinearity between the attributes. To explore the interactions effectively, future studies could use a blocked design, where a larger number of choice questions is generated, of which each respondent sees a randomly allocated subset [54].

Respondents were recruited by broad invitation to GP’s who had signed up to be part of a panel. It is possible that those who chose to participate in this study had a particular interest in infectious disease, or antibiotic and resistance. Their level of knowledge may therefore not be representative of the broader population of GPs. Similarly, the offer of an incentive to participate (although this is normal) may have appealed to a particular population among GPs, although it is not obvious that the presence of an incentive would have affected respondents’ answers in any particular direction in this study. Finally, the study was run before the COVID-19 pandemic, which led to a rapid shift to remote consultations; this change in practice may persist beyond the immediate lockdown restrictions. The effect of this may be reduced the certainty of prescribing in the absence of some physical examinations and face-to-face cues [55]. This could lead either to an increase in use of delayed prescription in response to that uncertainty, or to an increase in immediate prescriptions which could have important consequences for antimicrobial resistance. Future work is needed to identify any such changes in practice, and determine their effect on antibiotic consumption.

## 4. Methods

Study design, data collection and analysis followed good practice guidance for similar choice-based studies [56]. Ethical approval was granted by the University of Oxford Medical Sciences Interdivisional Research Ethics Committee (R58586/RE002). All respondents gave their informed consent before participating.

### 4.1. Defining Survey Attributes and Levels

Factors expected to influence GPs’ use of delayed prescription (termed ‘attributes’) were identified from a literature search (summarised in the Supplementary Material S1 Section 5). Eight attributes were selected from this long-list based on: (a) importance of rankings from a convenience sample of practicing GPs (*n* = 4); (b) face validity based on the clinical experience within the project team; and (c) policy relevance.

Levels for each attribute were determined from clinical guidelines [14,15], Cochrane reviews [8,41,57,58] and primary care studies [11,21,38,39,40], current NHS prescribing tools and support materials [36,59,60], and clinical expertise within the project team. Attributes, their levels, and rationale are shown in Table 1.

### 4.2. Choice Questions

Respondents (GPs) were asked to consider a consultation in which an adult patient presents with an RTI. In each choice question, they were presented with a profile describing the patient’s condition, and asked to choose between immediate or delayed prescription. To allow for the possibility that some respondents would have chosen not to prescribe antibiotics at all in some of the scenarios described, they were then offered the choice to prescribe no antibiotics, or remain with their original choice. The ‘no prescription’ option was not presented initially, to avoid losing information on our primary question regarding preferences for delayed prescription as an alternative to immediate prescription.

### 4.3. Survey and Experimental Design

The survey was presented on-line, in English (full survey text provided in Supplementary Material S1 Section 1). Respondents were provided with information about the survey and gave their informed consent to participate. They were given instructions on how to complete the survey, and an explanation of each of the attributes. The next section asked respondents to rank the attributes in order of importance to the prescribing context (with the attributes presented in randomised order), and to complete a practice choice question, which consisted of the attribute levels most likely to lead to an ‘immediate’ prescription.

Respondents then completed 15 choice questions; this is generally considered an acceptable number of questions in this type of survey [32,33,53,61,62]. All respondents saw the same 15 questions. Finally, respondents answered questions about themselves, their practice and their antibiotic prescribing. The survey was reviewed with GPs on the project team at all stages of its construction, to ensure clarity and medical accuracy.

The choice questions were produced using experimental design software, Ngene [63], to create an efficient design (that is, one that maximises the information available from respondents’ choices). Constraints were applied to avoid implausible scenarios (see Supplementary Material S1 Section 6). In line with recommended practice, an initial design was created, and used in a pilot sample (23 GPs recruited in the same way as for the main study). The choices from this pilot were used to optimise the design for the main study, but were not included in the final analysis. The most efficient design generated by the software was selected, following checks by clinical experts that none of the scenarios were implausible, nor were expected to lead to the same decision by all respondents.

A sample size estimate based on the standard errors predicted from the experimental design (52) indicated that at the target sample size of 180, the study would be able to detect coefficients of value 0.24 for the levels of the symptoms attribute, and 0.01 for the other attributes, at a two-sided significance level of 0.05 and with power of 80%.

### 4.4. Data Collection

Respondents were recruited from an existing panel of UK GPs curated by Medeconnect, a market research provider specialising in healthcare professionals. Quotas based on Medeconnect’s annual GP Omnibus study were used to recruit a study sample representative of UK GPs in terms of gender, age, country within the UK and practice size. Respondents who completed the survey received reward points equivalent to £20 in the form of vouchers, which is in line with standard practice of this provider for this type and length of survey. The data were collected in February and March 2019. No response rate could be calculated, as it was not known how many people would have seen the invitation to participate on the provider’s website.

### 4.5. Analysis

Data analysis was performed in Stata (v.15SE) [64]. Choices between delayed and immediate prescription were analysed using a mixed-effects logistic regression model, which models the log-odds of choosing delayed prescription as a linear combination of the attribute levels. This model was chosen because it allows for heterogeneity between respondents in their tendency to choose the delayed prescription (that is, it includes a random intercept per respondent), and can incorporate respondent characteristics directly as predictors. The coefficients represent the effect of one unit of the attribute on the log-odds of respondents choosing the delayed prescription. Cluster-robust standard errors were used throughout, to allow for the fact that each respondent contributes 15 responses. To assess whether the time and risk attributes could be appropriately represented as continuous variables with a linear relationship with the outcome, these attributes were also modelled as categorical variables (see Supplementary Material S1 Section 7).

For the second part of the question (including the no-prescription alternative) choices were modelled using a partial proportional ordered logit model (gologit2 command in Stata). This assumes the three possible outcomes have a natural order (immediate, delayed and no prescription) and models the probability of respondents choosing each outcome relative to the adjacent one in the hierarchy. The partial proportional model was chosen because it relaxes the assumption that each attribute has a consistent effect on the probability of choosing each category. The model generates two coefficients for each attribute: one for its effect on the probability of choosing an immediate prescription rather than delayed or no prescription, and one for its effect on the probability of choosing a prescription (either type) rather than no prescription. The two coefficients are tested to determine if they are statistically significantly different (Wald test, *p* < 0.05) and if so, they are retained as different coefficients.

To help interpret model coefficients, the average predicted probability of choosing each type of prescription for each level (the marginal predicted mean) was calculated, using the ‘margins’ command in Stata. This method sets the attribute to that level for all observations, keeping the other variables at their observed levels. The probability of choosing delayed prescription is then predicted for each observation using the regression model, and the mean probability calculated. This can also be expressed as the effect of one unit of the attribute on the probability of choosing the delayed prescription.

Models were compared using a measure of how much of the variability in responses was explained by the model (McKelvey and Zavoina Pseudo-R^2^ [65]) and measures of goodness-of-fit (the Akaike and Bayesian Information Criteria).

The dataset is available from the corresponding author on reasonable request.

## 5. Conclusions

Clinical features (symptoms, duration and comorbidities) are appropriately the most important factors for GPs in deciding between immediate, delayed and no antibiotic prescription. However, broader dissemination of the relevant clinical evidence for specific presentations may be helpful in supporting GPs to make greater use of delayed prescription. With patient opinion playing a role in the choice of prescription type, establishing a patient’s actual preference during the consultation may also help to reduce the number of immediate prescriptions. Extending consultation duration appears unlikely to increase use of delayed prescription.

## Figures and Tables

**Table 1:** Attributes and levels for the choice questions.

Attribute ^a^	Levels	Basis
Symptoms the patient is experiencing ^b^	1: Sore and red throat, and swollen lymph nodes in the neck 2: Productive cough and runny nose 3: Sore throat, swollen lymph nodes in the neck, pyrexia and purulent tonsils 4: Productive cough, pyrexia and pain on breathing	Two upper respiratory tract symptoms, and two lower, to allow exploration of differences in perception of ‘throat’ and ‘chest’ infections. Clinical guidelines [14,15], diagnostic criteria (such as FeverPAIN [35]) and practising clinicians were consulted to identify two plausible levels of severity for each, identified as ‘minor’ (1 and 2) and ‘serious’ (3 and 4) throughout this paper.
How long the person has had the symptoms when they see the primary care physician	3 days 7 days 10 days	Durations identified from literature [8], to cover a wide yet realistic range for RTIs.
Relevant comorbidities of the patient ^b^	1. None 2. One 3. Two or more	Reflects clinical guideline CG69 [14], which identifies comorbidities as a risk factor for developing complications, and particularly for acute cough, increasing risk with additional comorbidities or other patient factors.
Length of the consultation with the primary care physician	5 min 10 min 15 min	Proxy for quality of information exchange between primary care physician and patient. Levels represent plausible consultation durations; the longest consultation is intended to allow for use of tools such as TARGET patient leaflets [36] to explain treatment.
Patient opinion on taking antibiotics ^b^	1. Preference to have antibiotics 2. No preference expressed 3. Preference not to have antibiotics	Patient opinion can influence clinician choices [19,37]. Levels allow for patient preference in either direction, or neutral.
Risk of harm from not having antibiotic treatment straight away	1% 10% 20%	The GP’s judgement of the risk of harm, explained as symptom persistence or recurrence, or complications. Shown as a percentage, as a graphic, and also described in words (‘In 1 case out of every 100 like this, the patient would…’). Levels identified from literature. Rates of complications typically range from <1% to 2% for RTIs in primary care studies without antibiotics [11,38,39,40]. Symptom persistence at follow-up in the case of acute bronchitis ranges from 18% to 35% in meta-analyses depending on the measure [41], with a reconsultation rate of ~20% for non-resolution for RTIs [10,21], without antibiotics.
Risk of an adverse effect from taking antibiotics	1% 10% 20%	The GP’s judgement of the risk of adverse effect, explained as allergy, side effects, or future resistance. Shown in three formats, as above. Levels identified from literature and public information on rates of side effects and allergy [8,42].
How a delayed prescription would be provided ^b^	1: prescription plus advice to delay collection of antibiotics 2: post-dated prescription 3: collect prescription from the practice reception at a later date	Policy relevance: these formats have been tested in clinical trials [11] and referred to in guidelines [14], but there are no quantitative data on clinician preferences.

a Explanations of each attribute and its levels were provided in the survey (see Supplementary Materials S1 Section 1). b Categorical variable. Other attributes are treated as continuous variables.

**Table 2:** Respondent characteristics.

Respondent Characteristic		N (Percentage)	Quota (%) ^#^
Sex	Male	98 (54%)	56
Age (years)	39 and under	40 (22%)	26
	40–49	76 (42%)	41
	50–59	50 (28%)	24
	60 or over	15 (8%)	10
	Median age	46	
Country	England	152 (84%)	83
	Scotland	17 (9%)	10
	Wales	9 (5%)	4.5
	Northern Ireland	3 (2%)	2.5
Practice size	1–2500 patients	5 (3%)	4
	2501–5000	28 (15%)	15
	5001–7500	40 (22%)	20
	7501–10,000	35 (19%)	20
	10,001–12,500	32 (18%)	41
	12,501–15,000	14 (8%)	
	More than 15,000 patients	27 (15%)	
Role in practice	Partner	96 (53%)	
	Salaried GP	57 (31%)	
	Locum	28 (15%)	
Level of local deprivation *	High	49 (27%)	
	Medium	72 (40%)	
	Low	56 (31%)	
Practice’s level of antibiotic	Very low/Low	36 (20%)	
prescribing compared to	Average	100 (55%)	
similar practices *	Very high/high	35 (19%)	
Usual format of delayed prescription *	Standard prescription with recommendation to wait	145 (80%)	
	Post-dated prescription	23 (13%)	
	Electronic post-dated prescription	7 (4%)	
	Prescription available from practice at future date	4 (2%)	
	Other	2 (1%)	
RTI prescribing *: mean	An immediate antibiotic prescription (range)	31% (1 to 90%)	
percentage of patients	A delayed antibiotic prescription (range)	17% (0 to 85%)	
who leave with…	No antibiotic prescription (range)	52% (0 to 95%)	
Found the survey *	Very easy/easy/quite easy	90 (50%)	
	Neither easy nor difficult	52 (29%)	
	Very difficult/difficult/quite difficult	39 (21%)	

^#^ Based on annual GP omnibus survey, by Medeconnect, the online survey provider. * Self-reported.

**Table 3:** Effect of attributes on preferences for delayed prescription.

Attribute/Level		1. Attributes Only		2. Respondent Characteristics	
		Coefficient	95% CI	Coefficient	95% CI
Symptoms	Sore and red throat, and swollen lymph nodes in the neck (‘minor throat’)	3.17	2.48 to 3.86 *p* < 0.001	3.17	2.48 to 3.86 *p* < 0.001
	Productive cough and runny nose (‘minor chest’)	3.47	2.79 to 4.14 *p <* 0.001	3.47	2.79 to 4.14 *p <* 0.001
	Sore throat, swollen lymph nodes in the neck, pyrexia and purulent tonsils (‘serious throat’)	−0.90	−1.31 to −0.49 *p <* 0.001	−0.90	−1.31 to −0.49 *p <* 0.001
	Productive cough, pyrexia and pain on breathing (‘serious chest’) ^a^	0	-	0	-
Symptom duration	Per day longer	−0.33	−0.43 to −0.23 *p <* 0.001	−0.33	−0.43 to −0.23 *p <* 0.001
Relevant comorbidities	None ^a^	0	-	0	-
	One	0.05	−0.31 to 0.42 *p =* 0.769	0.06	−0.31 to 0.42 *p =* 0.762
	Two or more	−1.18	−1.64 to −0.72 *p <* 0.001	−1.18	−1.64 to −0.72 *p <* 0.001
Consultation length	Per minute longer	0.05	0.02 to 0.09 *p =* 0.003	0.05	0.02 to 0.09 *p =* 0.003
Patient opinion	Preference to have antibiotics	0.39	−0.72 to −0.05 *p =* 0.022	−0.39	−0.72 to −0.05 *p =* 0.023
	No preference expressed ^a^	0	-	0	-
	Preference not to have antibiotics	0.33	0.05 to 0.60 *p =* 0.020	0.33	0.05 to 0.60 *p =* 0.020
Risk of harm from not starting antibiotics	Per 1% higher	−0.13	−0.17 to −0.10 *p <* 0.001	−0.13	−0.17 to −0.10 *p <* 0.001
Risk of adverse effect from taking antibiotics	Per 1% higher	0.03	0.01 to 0.05 *p =* 0.001	0.03	0.01 to 0.05 *p =* 0.001
Format of the delayed prescription	Advice to delay ^a^	0	-	0	-
	Post-dated prescription	−0.03	−0.37 to 0.31 *p =* 0.872	−0.03	−0.37 to 0.31 *p =* 0.872
	Collect from practice	−0.43	−0.82 to −0.08 *p =* 0.016	−0.45	−0.82 to −0.08 *p =* 0.016
Self-reported prescribing behaviour: percent immediate prescriptions for RTI				−0.02	−0.04 to −0.01 *p =* 0.002
Intercept		2.23	1.49 to 2.97 *p <* 0.001	3.44	2.46 to 4.42 *p <* 0.001
Var(intercept) ^b^		1.57	0.96 to 2.57	1.41	0.88 to 2.28
Pseudo R^2^: attributes only		0.61		0.62	
Pseudo R^2^: attributes and respondent-level effect		0.65		0.66	
Akaike Information Criterion		1955		1943	
Bayesian Information Criterion		2043		2037	

^a^ Reference level for the categorical variables. The coefficient for each level shows the effect of that level on the log odds of choosing delayed prescription, relative to the reference level. ^b^ Variance of the random intercept. This term reflects the unexplained variation between respondents in their tendency to choose the delayed prescription after accounting for explanatory variables listed in the table. CI, confidence interval.

## Supplementary Materials

The following are available online at https://www.mdpi.com/2079-6382/9/9/608/s1, Supplementary Materials S1.

## Abbreviations

abx	antibiotics
GP	general practitioner
NHS	National Health Service
NICE	National Institute for Health and Care Excellence
RTI	respiratory tract infection
