# Supplementary material for: Delayed Antibiotic Prescription by General Practitioners in the UK: A Stated-Choice Study
Source: Antibiotics (Basel). 2020 Sep 16;9(9):608. doi: 10.3390/antibiotics9090608 (PMC7558347; doi:10.3390/antibiotics9090608)
Supplement: Supplementary file 1 [file antibiotics-09-00608-s001.pdf]

SUPPLEMENTARY MATERIAL

Delayed antibiotic prescription by general practitioners in the UK: a stated-choice study

Morrell, L et al, on behalf of the STEP-UP team

Contents

1. Survey instrument

2. Marginal effects of the attributes on the probability of choosing the delayed prescription (mixed effect logit model)

3. Comparison of mixed-effect and ordered logistic regression models

4. Predicted probabilities of choosing immediate, delayed or no prescription (ordered logit model)

5. Literature search and rationale for attribute selection

6. Design constraints

7. Modelling the continuous variables as categorical (mixed effect logit model)

SECTION 1. Survey instrument

A survey on attitudes to antibiotic prescribing in general practice

Doctors.net.uk invites you to take part in a short survey commissioned by an academic researcher to investigate your attitude to antibiotic prescribing decisions in general practice.

The aim of this study is to learn more about the factors that are important to GPs when deciding how to treat patients who they think might need an antibiotic. The results will help to inform antibiotic prescribing policies for the NHS in the future. We would like to invite you as a GP to participate in our online survey.

In this survey, we will show you a series of hypothetical situations. We will ask you to make a decision about antibiotic treatment for the patient described in each situation.

The survey should take up to 20 minutes to complete and all members completing the survey in full will receive 4000 eSR points. We would like you to answer all of the questions. To help you to answer the questions we have provided some background information for you to read at the start of the survey.

Please note that your participation is voluntary. You may withdraw at any point during the questionnaire for any reason, before submitting your answers, simply by closing your computer's browser window. However, we are only able to award points to participants who complete the full survey.

The survey is being run by the Nuffield Department of Population Health at the University of Oxford. The lead researcher is Dr. Liz Morrell. This project has been reviewed by, and received ethics clearance through, the University of Oxford Central University Research Ethics Committee [R58586/RE002].

Please read the following text, which further explains the key aspects of this research:

- I understand that this research is commissioned by an academic researcher and is being carried out within the code of conduct of the Market Research Society and the British Healthcare Business Intelligence Association
- Doctors.net.uk will comply with all UK laws protecting your personal data and the British Healthcare Business Intelligence Association and Market Research Society guidelines
- The research is not intended to be promotional and any information presented is done so solely to explore reactions to such information
- Your responses will be totally anonymous and confidential
- The aggregated findings of this research may be published in academic publications, however at no stage will it be possible to identify any participants

Doctors.net.uk is the data controller with respect to your personal data and, as such, will determine how your personal data is used. Please see Doctors.net.uk's privacy notice here. Doctors.net.uk will share only fully anonymised data with the University of Oxford, for the purposes of research.

All results will be anonymised in accordance with Doctors.net.uk's zero-tolerance privacy policy and the Market Research Society Code of Conduct. The anonymised data will be stored on secure networks at the University of Oxford, and archived securely at the end of the project.

What if there is a problem?
If you wish to contact us about this survey, here are our contact details.

Researcher: Peter Constable   Email: SurveyHelp@mess.doctors.org.uk
Contact reference for inclusion in the email subject: Survey 01206970

If we cannot resolve your question, we will contact the lead researcher and work with her to deal with your concern. If you remain unhappy or wish to make a formal complaint, we will provide you with contact details for the Chair of the Research Ethics Committee at the University of Oxford.

Please confirm that you have read and understood this information, and agree to take part in the survey

Yes
No   **THANK AND CLOSE**

[screener questions for quotas]

1. How old are you?
   [write in number]

2. What is your gender?
   Male   Female   Other   Prefer not to say

3. In which part of the UK do you currently practice?

4. Thinking about the number of registered patients, how big is the practice where you work most often?
   Less than 5000   5001-10000   10001-15000   greater than 15000

Thank you for providing that information.

This survey is about the management of respiratory tract infections (RTIs) in general practice.

Current guidelines for antibiotic use in RTIs (for example, NICE clinical guideline 69) allow for the use of one of three antibiotic prescribing strategies: immediate prescribing, delayed or ‘back-up’ prescribing, or no prescribing. We want to understand how GPs decide whether to give an immediate or a delayed prescription to a patient with an RTI who they believe might need antibiotics.

We are interested in your views about what factors are important when deciding on the treatment approach for a patient with an RTI. Our results will help inform future antimicrobial stewardship practices and clinical guidelines.

The next page provides some information on the survey, and how to complete it.

HOW TO COMPLETE THIS SURVEY

For this survey, we would like you to consider the following hypothetical scenario:

> *An adult patient has presented with a respiratory tract infection. You think that the patient might need antibiotics, and you now need to decide whether to prescribe immediate antibiotics, or provide a delayed prescription for antibiotics that the patient can take later if necessary.*

In the survey, we will show you a series of situations and ask you two questions about each. First, we will ask you to decide whether to give the patient an immediate or delayed prescription for antibiotics. We appreciate that prescribing preferences vary, and in some situations you might prefer not to prescribe antibiotics. We will ask if that is the case in the second question. Regardless of whether you would prefer not to prescribe antibiotics, we would still like you to answer the first question, and decide between an immediate or delayed prescription in each situation.

Each situation is made up of eight features, describing the patient's condition, and the discussion during your consultation. There are three or four possible options for each feature, and the options that are presented to you will vary in each situation. Detailed descriptions of each feature and the possible options are provided on the following pages. Please read these descriptions carefully, then answer all of the questions that follow.

THANK YOU FOR YOUR HELP

Feature 1

Patient's symptoms and physical signs

In each situation, the symptoms will be one of the following four options:

- Sore and red throat, and swollen lymph nodes in the neck
- Sore throat, swollen lymph nodes in the neck, pyrexia, and purulent tonsils
- Productive cough and runny nose
- Productive cough, pyrexia, and pain on breathing

Feature 2

Duration of the patient's symptoms

This describes how long the patient has had their current symptoms, before their consultation with you. In each situation, this will be one of the following three periods:

- 3 days
- 7 days
- 10 days

Feature 3

Relevant comorbidities or complicating factors

Relevant comorbidities could include, for example, heart or lung conditions, another chronic disease, a recent hospitalisation, or the patient being aged over 65.

In each situation, the patient will have:

- No relevant comorbidities
- One relevant comorbidity
- Two or more relevant comorbidities

Feature 4

Length of time you have available to spend with this patient

In each situation, the length of time spent with the patient will be one of the following three options:

- 5 minutes
- 10 minutes
- 15 minutes

Feature 5

Opinions expressed by the patient about antibiotic treatment

In each situation, the opinions expressed by the patient will be one of the following:

- Patient has expressed a preference to have antibiotics
- Patient has not expressed any preference relating to antibiotics
- Patient has expressed a preference not to have antibiotics

Feature 6:

Likelihood of harm from <u>not having</u> immediate antibiotic treatment

This harm might be persistence or recurrence of presenting symptoms, or complications, all of which may lead to a re-consultation.

You should assume that your judgement of this probability is based on your clinical experience, local or national guidelines, and evidence from clinical studies. Your judgement will include patient factors not reflected in the profile so you should accept the risk figures given as accurate.

In each situation, the likelihood that the patient will experience harm from not having antibiotics will be one of three options, which are illustrated in the diagrams below:

- <u>Unlikely</u>: in 1 case out of every 100 like this, the patient will experience persistent or recurrent symptoms, or complications

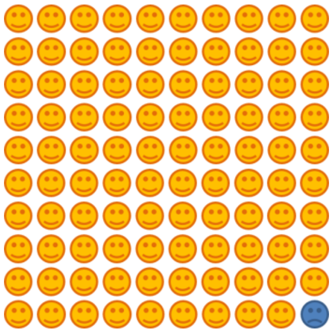

- <u>Somewhat likely</u>: in 10 cases out of every 100 like this, the patient will experience persistent or recurrent symptoms, or complications

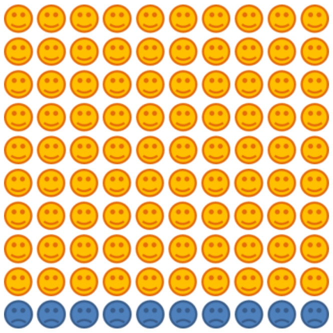

- <u>Likely</u>: in 20 cases out of every 100 like this, the patient will experience persistent or recurrent symptoms, or complications

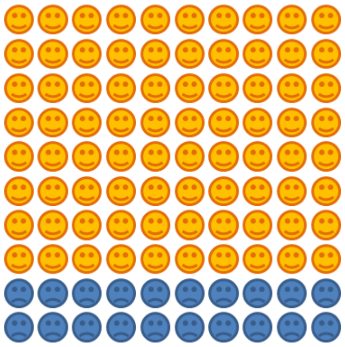

Feature 7

Likelihood of an adverse effect from having antibiotic treatment

An adverse effect might be a gastro-intestinal side effect, an allergic reaction, or a future antibiotic-resistant infection. You should assume that your judgement of this probability is based on clinical experience, local or national guidelines, and evidence from clinical trials for the antibiotic(s) you are considering prescribing.

In each situation, the likelihood that the patient will experience an adverse event from taking antibiotics will be one of three options, which are illustrated in the diagrams below:

- Unlikely: in 1 case out of every 100 like this, the patient will experience an adverse effect from antibiotic treatment

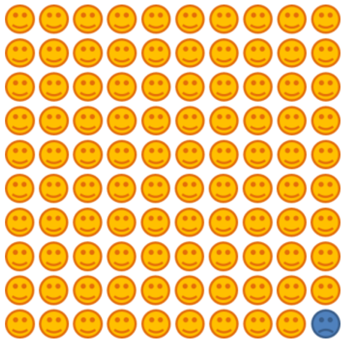

- Somewhat likely: in 10 cases out of every 100 like this, the patient will experience an adverse effect from antibiotic treatment

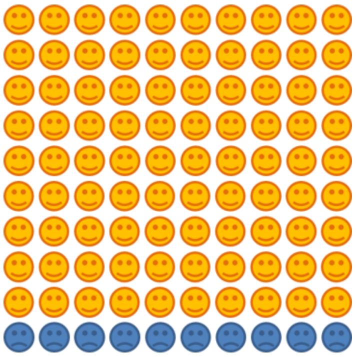

- Likely: in 20 cases out of every 100 like this, the patient will experience an adverse effect from antibiotic treatment

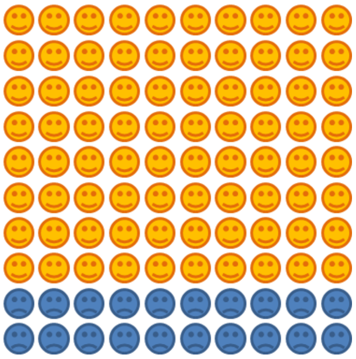

Feature 8

How your practice would issue a delayed prescription

Practices may have different ways of issuing delayed prescriptions. They differ in how much control you have over when the patient collects the medication, and convenience for the patient. In each situation, please assume you have one of the following three mechanisms available to you:

- <u>Give a prescription with recommendation to delay collection</u>. You would hand the patient a prescription, but advise them only to collect the antibiotics if their symptoms do not start to resolve after a specified number of days.
- <u>Give a post-dated prescription.</u> You would hand the patient a prescription showing a date several days in the future. The pharmacy would only be able to dispense the antibiotics on or after that date.
- <u>Prescription available from the practice at a future date.</u> You would not hand the patient a prescription during the consultation. You would explain that if their symptoms do not start to resolve after a specified number of days, they can return to the practice and collect the prescription from the receptionist.

[SUMMARY TABLE]

The table below summarises the eight features and the different options that may be presented to you in each situation.

FEATURE	POSSIBLE OPTIONS
Patient's symptoms and signs	• Sore and red throat, and swollen lymph nodes in the neck<br>• Sore throat, swollen lymph nodes in the neck, pyrexia, and purulent tonsils<br>• Productive cough and runny nose<br>• Productive cough, pyrexia, and pain on breathing
Duration of symptoms	• 3 days<br>• 7 days<br>• 10 days
Patient's comorbidities	• No relevant comorbidities<br>• One relevant comorbidity<br>• Two or more relevant comorbidities
Length of time to spend with the patient	• 5 minutes<br>• 10 minutes<br>• 15 minutes
Patient's opinions	• Patient has expressed a preference to have antibiotics<br>• Patient has not expressed any preference relating to antibiotics<br>• Patient has expressed a preference not to have antibiotics
Likelihood of harm from not having immediate antibiotics	• Unlikely: in 1 case out of every 100 like this, the patient will experience persistent or recurrent symptoms, or complications<br>• Somewhat likely: in 10 cases out of every 100 like this, the patient will experience persistent or recurrent symptoms, or complications<br>• Likely: in 20 cases out of every 100 like this, the patient will experience persistent or recurrent symptoms, or complications
Likelihood of an adverse effect from having antibiotics	• Unlikely: in 1 case out of every 100 like this, the patient will experience an adverse effect<br>• Somewhat likely: in 10 cases out of every 100 like this, the patient will experience an adverse effect<br>• Likely: in 20 cases out of every 100 like this, the patient will experience an adverse effect
How your practice would issue a delayed prescription	• Give a prescription with recommendation to delay collection<br>• Give a post-dated prescription<br>• Prescription available from the practice at a future date

[RANKING EXERCISE]

We would now like to know which of these features are most important to you, when you are making antibiotic prescribing decisions for RTIs.

Please consider all eight features and then rank them below. Rank the most important feature as 1, down to the least important as 8.

Drag and drop the items in order of importance, placing the most important at the top and the least important at the bottom

[Randomised presentation order]

Patient’s symptoms and signs
Duration of symptoms
Patient’s comorbidities
Length of time to spend with the patient
Patient’s opinions
Likelihood of harm arising from not having immediate antibiotics
Likelihood of an adverse effect from having antibiotics
How your practice would issue a delayed prescription

[PRACTICE QUESTION]

Now we would like you to complete a practice choice question, as an example of the questions you will see in the main survey.

Please consider the following scenario:

> *An adult patient has presented with a respiratory tract infection. You think that the patient might need antibiotics, and you now need to decide whether to prescribe immediate antibiotics, or provide a delayed prescription for antibiotics that the patient can take later if necessary.*

The situation is described below.

Patient's symptoms and signs	Productive cough, pyrexia, and pain on breathing
Duration of symptoms	3 days
Patient's comorbidities	2 or more relevant comorbidities
Length of time to spend with patient	5 minutes
Patient's opinions	Preference to have antibiotics
Likelihood of harm arising from not having immediate antibiotics	Likely (20/100)
Likelihood of an adverse effect from having antibiotics	Unlikely (1/100)
How your practice would issue a delayed prescription	Prescription available from the practice at a future date

In this situation which would you give the patient:

An immediate antibiotic prescription ☐
OR
A delayed antibiotic prescription ☐

If we had offered the option of not prescribing antibiotics for this patient, would you have selected:

An antibiotic prescription, as chosen above ☐
OR
No antibiotic prescription ☐

[MAIN QUESTIONS]

Thank you for completing the ranking exercise and practice question.

Now we would like you to complete the main part of the survey.

We are going to describe 15 situations.

In all of them, we will ask you to consider the same scenario, of an adult patient with an RTI, as in the practice question. However, the characteristics of the patient and the consultation will be different each time.

Please indicate for each situation, whether you would give the patient an **immediate prescription** or a **delayed prescription** for antibiotics.

QUESTION 1

Please consider the following scenario:

An adult patient has presented with a respiratory tract infection. You think that the patient might need antibiotics, and you now need to decide whether to prescribe immediate antibiotics, or provide a delayed prescription for antibiotics that the patient can take later if necessary.

The situation is described below.

Patient's symptoms and signs	Sore and red throat, and swollen lymph nodes in the neck
Duration of symptoms	10 days
Patient's comorbidities	Two or more relevant comorbidities
Length of time to spend with patient	10 minutes
Patient's opinions	Preference to have antibiotics
Likelihood of harm arising from not having immediate antibiotics	Unlikely (1/100)
Likelihood of an adverse effect from having antibiotics	Somewhat likely (10/100)
How your practice would issue a delayed prescription	Prescription available from the practice at a future date

In this situation which would you give the patient:

An immediate antibiotic prescription ☐
OR
A delayed antibiotic prescription ☐

If we had offered the option of not prescribing antibiotics for this patient, would you have selected:

An antibiotic prescription, as chosen above ☐
OR
No antibiotic prescription ☐

(similarly for questions 2-15)

[RANKING EXERCISE – PART TWO]

Thank you for completing the main part of the survey.

Now we would like you to complete the ranking exercise again to see if your opinions have changed after answering these questions.

Please consider how important the eight features are to you, and then rank them below. Rank the most important feature as 1, down to the least important as 8.

Drag and drop the items in order of importance, placing the most important at the top and the least important at the bottom

Patient's symptoms and signs
Duration of symptoms
Patient's comorbidities
Length of time to spend with the patient
Patient's opinions
Likelihood of harm arising from not having immediate antibiotics
Likelihood of an adverse effect from having antibiotics
How your practice would issue a delayed prescription

[RESPONDENT CHARACTERISTICS]

Now we would like to ask some questions about you.

All of the information that you provide will help us in our analysis, and all of your details will remain confidential. If you do not wish to answer some of these questions you do not have to – you can just skip the question.

The first questions are about your responses to the scenarios.

1. When you were answering the choice questions, what 'adverse effect(s)' from having antibiotics were you predominantly considering?
   [free text]

2. The survey questions assumed that you could judge the likelihood of patients experiencing adverse effects from antibiotic treatment. How easy or difficult was it to imagine you could judge this for:
   side effects
   allergic reactions
   future antibiotic resistance

   [5-point scale for each: Very easy – Easy – Neither easy nor difficult – Difficult – Very Difficult]

   Are there any further comments you would like to make regarding judgement of the likelihood of adverse effects from antibiotic treatment?
   [free text]

Now we would like to ask some questions about your practice.

3. Which of the following options best describes your role in the practice where you work most often?
   Partner   Salaried GP   Locum   GP trainee   Other (please write in [text])

4. Relative to other areas in the UK, how would you describe the level of deprivation in the area where you work most often?
   High   Medium   Low   I don't know

5. Compared to similar practices, how would you describe the level of antibiotic prescribing in the practice where you work most often?
   Very low   Low   Average
   High   Very high   I don't know

6. What guidelines, if any, do you follow for antibiotic prescribing in RTIs? (please select all that apply)
   NICE
   Department of Health and Social Care
   Royal College of GPs
   CCG
   Practice's own
   Public Health England
   Health Protection Scotland
   Public Health Agency Northern Ireland
   Public Health Wales
   Other (please write in: [text])

7. Please estimate what percentage of your patients with an RTI leave the consultation with:
    - An immediate antibiotic prescription [write in 1-100]
    - A delayed antibiotic prescription [write in 1-100]
    - No antibiotic prescription [write in 1-100]

8. In the practice where you most often work, how are delayed antibiotic prescriptions usually issued? [select one]
    - Standard prescription with recommendation to delay collection
    - Post-dated prescription
    - Electronic prescription with delayed collection date
    - Prescription available from the practice at a future date
    - Other (please specify) [free text]

9. We would like to end this survey by asking about how you see yourself. There are 10 statements below. Please indicate how well each of these statements describes your personality.
[This question is not mandatory]

    “I see myself as someone who is reserved.”
    “I see myself as someone who is generally trusting.”
    “I see myself as someone who tends to be lazy.”
    “I see myself as someone who is relaxed, handles stress well.”
    “I see myself as someone who has few artistic interests.”
    “I see myself as someone who is outgoing, sociable.”
    “I see myself as someone who tends to find fault with others.”
    “I see myself as someone who does a thorough job.”
    “I see myself as someone who gets nervous easily.”
    “I see myself as someone who has an active imagination.”

    [Response options]

    Agree strongly | Agree a little | Neither agree nor disagree | Disagree a little | Disagree strongly

10. Finally, are you generally a person who is fully willing to take risks or do you try to avoid taking risks? Please indicate on the scale below, where 0 means “not at all prepared to take risks” and 10 means “fully prepared to take risks”. [This question is not mandatory.]

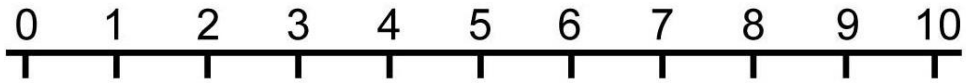

Not at all prepared to take risks

Fully prepared to take risks

11. How easy or difficult did you find the questions in this survey?
    [Likert scale 1-7, 1 = very easy, 7 = very difficult]

12. Are there any further comments that you would like to make regarding this survey?
    [free text]
    [not mandatory]

You have reached the end of the survey. Thank you for taking the time to participate.

SECTION 2

Effect of attributes on the probability of respondents choosing the delayed prescription

Attribute/level		Coefficient	95% CI	Effect on probability [a]	95% CI
Symptoms	Sore and red throat, and swollen lymph nodes in the neck ('minor throat')	3.17	2.48 to 3.86 p<0.001	0.39	0.32 to 0.46
	Productive cough and runny nose ('minor chest')	3.47	2.79 to 4.14 p<0.001	0.41	0.35 to 0.47
	Sore throat, swollen lymph nodes in the neck, pyrexia and purulent tonsils ('serious throat')	-0.90	-1.31 to -0.49 p<0.001	-0.14	-0.21 to -0.08
	Productive cough, pyrexia and pain on breathing ('serious chest') [b]	0	-	-	-
Symptom duration	Per day longer	-0.33	-0.43 to -0.23 p<0.001	-0.03	-0.04 to -0.02
Relevant comorbidities	None [b]	0	-	-	-
	One	0.05	-0.31 to 0.42 p=0.769	0.01	-0.03 to 0.04
	Two or more	-1.18	-1.64 to -0.72 p<0.001	-0.12	-0.16 to -0.07
Consultation length	Per minute longer	0.05	0.02 to 0.09 p=0.003	0.005	0.002 to 0.008
Patient opinion	Preference to have antibiotics	-0.39	-0.72 to -0.05 p=0.022	-0.04	-0.08 to -0.01
	No preference expressed [b]	0	-	-	-
	Preference not to have antibiotics	0.33	0.05 to 0.60 p=0.020	0.03	0.01 to 0.06
Risk of harm from not starting abx	Per 1% higher	-0.13	-0.17 to -0.10 p<0.001	-0.01	-0.02 to -0.01
Risk of adverse effect from taking abx	Per 1% higher	0.03	0.01 to 0.05	0.003	0.001 to 0.005

			p=0.001		
Format of the delayed prescription	Advice to delay [b]	0	-	-	-
	Post-dated prescription	-0.03	-0.37 to 0.31<br>p=0.872	-0.003	-0.03 to 0.03
	Collect from practice	-0.43	-0.82 to -0.08<br>p=0.016	-0.04	-0.08 to -0.01

a The effect of the attribute on the probability of respondents choosing the delayed prescription. For categorical attributes, this is the change in probability when the attribute was set at this level, compared to the reference level. For continuous attributes, this is the change in probability for a one-unit increase in the attribute.

b Reference level for the categorical variables. The coefficient for each level shows the effect of that level on the likelihood of choosing delayed prescription, relative to the reference level

abx antibiotics

SECTION 3

Table showing coefficients from mixed logit and generalised ordered logit side-by-side for ease of comparison (coefficient and 95% confidence interval)

		**Mixed logit**	**Generalised ordered logit**	
Attribute/level		Delayed vs Immediate	None or delayed vs Immediate	None vs Delayed or Immediate
Symptoms	Sore and red throat, and swollen lymph nodes in the neck ('minor throat')	3.17 (2.48 to 3.86)	2.48 (2.09 to 2.87)	2.04 (1.71 to 2.36)
	Productive cough and runny nose ('minor chest')	3.47 (2.79 to 4.14)	3.02 (2.46 to 3.58)	2.48 (2.10 to 2.85)
	Sore throat, swollen lymph nodes in the neck, pyrexia and purulent tonsils ('serious throat')	-0.90 (-1.31 to -0.49)	-0.77 (-1.03 to -0.50)	-0.77 (-1.03 to -0.50)
	Productive cough, pyrexia and pain on breathing ('serious chest') [a]	0	0	0
Symptom duration	Per day longer	-0.33 (-0.43 to -0.23)	-0.26 (-0.31 to -0.21)	-0.26 (-0.31 to -0.21)
Relevant comorbidities	None [a]	0	0	0
	One	0.05 (-0.31 to 0.42)	-0.32 (-0.56 to -0.07)	-0.32 (-0.56 to -0.07)
	Two or more	-1.18 (-1.64 to -0.72)	-1.14 (-1.43 to -0.85)	-1.14 (-1.43 to -0.85)
Consultation length	Per minute longer	0.05 (0.02 to 0.09)	0.02 (-0.01 to 0.05)	0.02 (-0.01 to 0.05)
Patient opinion	Preference to have antibiotics	-0.39 (-0.72 to -0.05)	-0.34 (-0.69 to 0.01)	0.26 (-0.06 to 0.59)
	No preference expressed [a]	0	0	0
	Preference not to have antibiotics	0.33 (0.05 to 0.60)	0.11 (-0.12 to 0.33)	0.11 (-0.12 to 0.33)
Risk of harm from not starting abx	Per 1% higher	-0.13 (-0.17 to -0.10)	-0.09 (-0.12 to -0.06)	-0.12 (-0.15 to -0.10)
Risk of adverse effect from taking abx	Per 1% higher	0.03 (0.01 to 0.05)	0.01 (0.00 to 0.02)	0.01 (0.00 to 0.02)
Format of the delayed prescription	Advice to delay [a]	0	0	0
	Post-dated prescription	-0.03 (-0.37 to 0.31)	-0.18 (-0.43 to 0.08)	-0.18 (-0.43 to 0.08)
	Collect from practice	-0.43 (-0.82 to -0.08)	-0.34 (-0.60 to -0.07)	-0.34 (-0.60 to -0.07)
Intercept		2.23 (1.49 to 2.97)	2.79 (2.02 to 3.56)	1.65 (0.88 to 2.42)

a Reference level for the categorical variables. The coefficient for each level shows the effect of that level on the likelihood of choosing delayed prescription, relative to the reference level

abx   antibiotics

SECTION 4

Graphs showing the predicted probability of choosing immediate, delayed or no prescription, for each level of each of the categorical variables. Predictions are based on the ordered logit model.

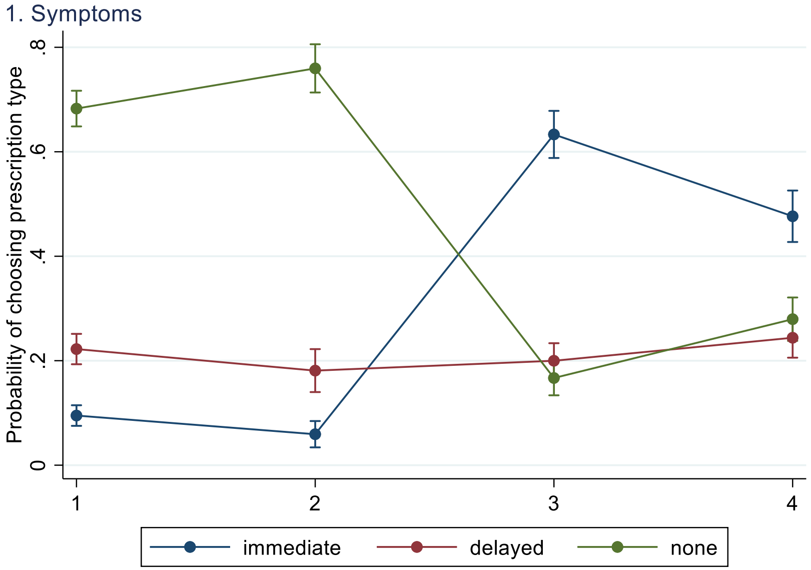

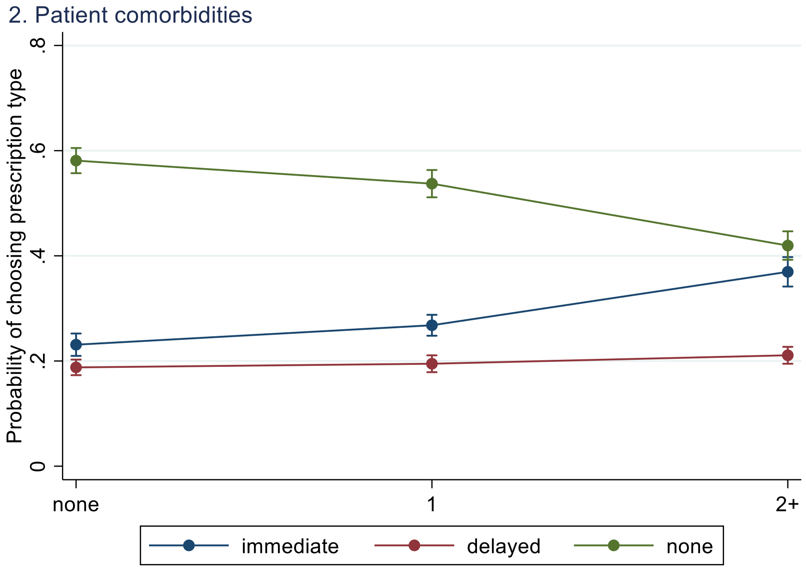

1 minor throat: sore and red throat, and swollen lymph nodes in the neck

2 minor chest: productive cough and runny nose

3 serious throat: sore throat, swollen lymph nodes in the neck, pyrexia and purulent tonsils

4 serious chest: productive cough, pyrexia and pain on breathing

none: no relevant comorbidities

1: one relevant comorbidity

2+: two or more relevant comorbidities

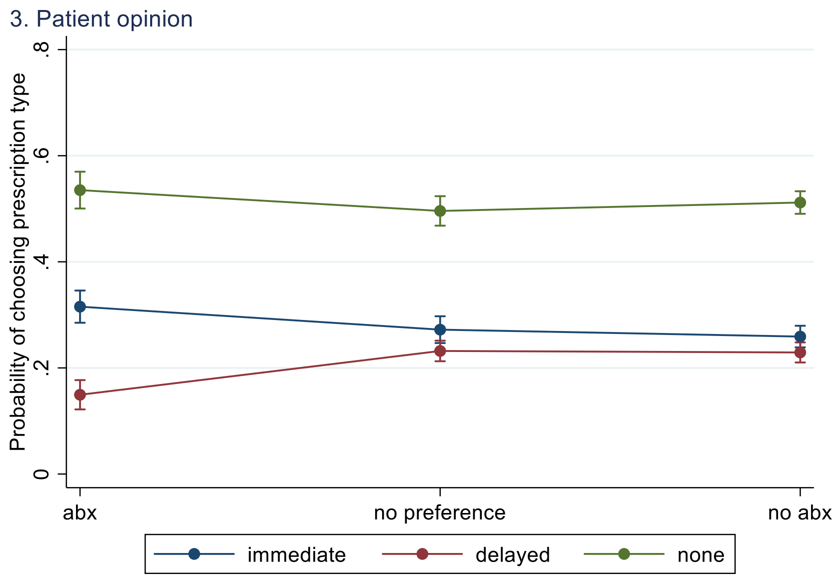

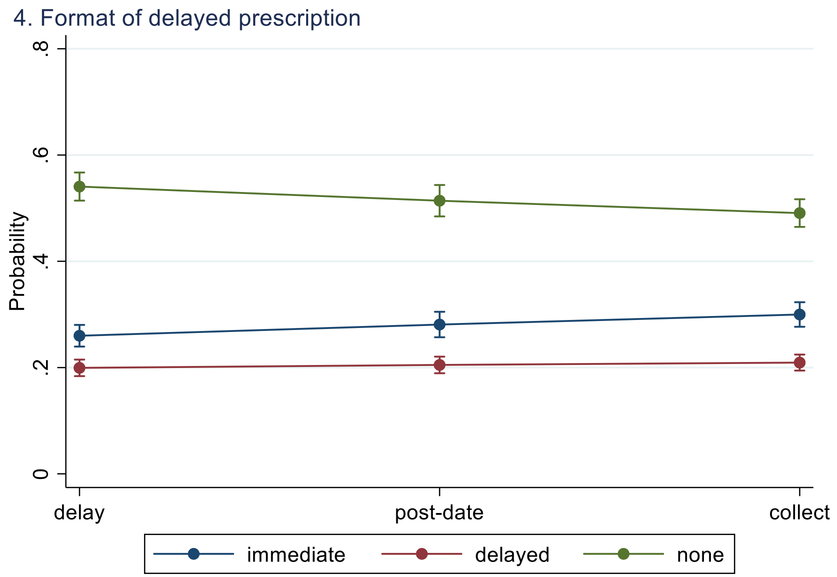

abx: expressed a preference to have antibiotics

no abx: expressed a preference not to have antibiotics

delay: advice to delay starting antibiotics

post-date: prescription has a date in the future

collect: collect prescription from practice reception

SECTION 5. Literature search and rationale for attribute selection

The attribute long-list was developed from a structured literature review, conducted to inform a series of choice studies in different contexts, one of which was this choice study. The overall aim was to generate a long-list of attributes that could potentially influence clinicians, patients, or members of the public, in giving, seeking or stopping antibiotic treatment for any condition.

Searches were restricted to studies in humans, and used the search syntax:

> (antibacterial OR anti-bacterial OR antibiotic* OR anti-infective OR antimicrobial* OR anti-microbial* OR AMR) AND (preference* OR DCE OR conjoint* OR best-worst* OR BWS OR discrete choice*)

Databases searched:

	**PubMed**	**Embase**	**Econlit**	**PsychInfo**
**Date range searched**	01/01/2005-12/02/2017	01/01/2005-12/02/2017	01/01/2005-12/02/2017	01/01/2005-13/07/2017

The literature search was undertaken in July 2017, and identified 3,066 papers. After removing duplicates, screening titles and abstracts, and then assessing full-text papers for eligibility, 89 papers met the inclusion criteria. In addition, 23 papers were identified from other sources (for example, papers that were already known to the study team). Overall, 112 papers were included.

116 potential attributes were identified in these papers. Attributes that might be important to general practitioners (GPs) in the context of this choice study were identified through discussion within the study team, which included 4 practicing GPs and a pharmacist. Related ideas were collapsed into single attributes where necessary (for example, the ‘symptoms’ attribute resulted from collapsing an attribute ‘severity of symptoms’ with specific symptoms such as ‘colour of nasal discharge’ and ‘abnormal lung sounds’). 17 such attributes were then scored for importance by a convenience sample.

Table S1: Summary of attributes as scored by convenience sample of GPs

	Attributes	**GPs (N=4)**	
		**Mean score**	**Rank**
1.	Symptoms	7.8	1=
2.	Premorbid condition of patient	7.8	1=
3.	Risk of significant harm from not giving antibiotic treatment	7.5	3=
4.	Recommendation from guidelines, literature or local protocol	7.5	3=
5.	Degree of benefit from antibiotics	7.3	5
6.	Length of illness	6.5	6=
7.	Risk of antibiotic resistance developing	6.5	6=
8.	Whether antibiotics are indicated by a diagnostic test	6.3	8
9.	Patient’s age	6.0	9
10.	Length of consultation time available	5.5	10=
11.	Information on resistance patterns/rates (e.g. from antibiogram)	5.5	10=
12.	Risk of significant harm from giving antibiotic treatment	5.3	12
13.	Number of days off work due to sickness	4.8	13
14.	Pressure from patient or family to prescribe antibiotics	4.0	14=

15.	Probability that patient might acquire a bacterial infection, or that infection may recur	4.0	14=
16.	Financial incentives	3.0	16
17.	Cost of antibiotics	2.0	17

We aimed to describe the choice situations using 6-8 attributes, to be acceptable to respondents without making choices excessively complex. Further, we aimed to maximise overlap between this work and a related study among the general public, for comparability and to identify potential differences between patients and clinicians. This meant it was possible for some attributes to be excluded from this study, despite a high importance ranking, if they were less important to the public.

The four highest ranked attributes were initially selected for inclusion. However, on further review of the guidelines, we noted that these already incorporate symptoms and comorbidities as part of the decision process. An attribute indicating consistency with guidelines would therefore need to be aligned with the clinical features described, and as such would add limited additional information to the study (that is, it could not be independent of the symptoms and comorbidities). We therefore did not select this attribute.

'Degree of benefit from taking antibiotics' was important to GPs, and to the general public. However, capturing this as an attribute appeared problematic, as GPs will have their own views on likely benefit. Telling respondents what the degree of benefit is in an attribute-level did not therefore seem feasible. The attribute 'Risk of harm from not giving antibiotics' captures a similar concept (the degree of benefit is in avoiding the harms from not taking antibiotics), which was more readily captured in a numerical attribute derived from trial data. Hence we chose to use the attribute framed in terms of the risk of harm.

Length of illness was ranked sixth, was also important to members of the public, and is used in some diagnostic algorithms (e.g. FeverPAIN criteria for sore throat), and so was included.

Risk of antibiotic resistance developing had the same rank as length of illness. However, comments from respondents suggested this would should be couched at a personal rather than societal level (that is, the risk of this patient developing a resistant infection in the future), which realistically is not known. We therefore chose to incorporate this feature within 'risk of harm from giving antibiotic treatment', with 'harm' described as the risk of side effects, allergy, and resistance.

Diagnostic testing was important to both groups; however, there is no good test for RTIs in common use in primary care in the UK, so it was felt this attribute was unrealistic and it was not selected.

Although 'Length of consultation time' only got a moderately high rank, we felt it was important to include this for face validity. It takes time to explain what a delayed prescription is and why it is being given.

Attributes relating to patient characteristics (such as age, socioeconomic status) were potentially of interest – for example, one respondent commented that time taken off work could be a factor if their patient was of low socio-economic status and missing work could have important consequences. However, there are multiple possible variables we could have considered, and somewhat arbitrary choices would be needed to describe relevant patient profiles in a single attribute. We therefore chose to keep patient characteristics constant across scenarios, and acknowledge that further work may be needed on this question.

The format of delivering the delayed prescription was not part of this preliminary survey. It was included for policy relevance; the formats have been tested in clinical trials and referred to in guidelines, but there are no quantitative data on patient preferences.

Reference: FeverPAIN clinical score

Little P, Stuart B, Hobbs FDR, Butler CC, Hay AD, Campbell J, et al. Predictors of suppurative complications for acute sore throat in primary care: prospective clinical cohort study. BMJ : British Medical Journal. 2013;347:f6867. Scoring tool available at https://ctu1.phc.ox.ac.uk/feverpain/index.php

SECTION 6. Design constraints

Constraints on which levels of specific attributes could not appear together were suggested by reviewing designs run without any constraints, and checking for implausible combinations of levels.

1. If the level for Symptoms was ‘Sore throat, swollen lymph nodes in the neck, pyrexia, and purulent tonsils’ or ‘Productive cough, pyrexia, and pain on breathing’ then the following attribute levels were **not** permitted:

    - Duration: 10 days. It is unlikely that a patient would have been suffering a fever for such an extended period without seeking medical advice – or in fact would have had a fever for such a length of time without the condition either resolving or progressing.
    - Appointment: 5 minutes. It is implausible that even the busiest GP would spend only 5 minutes with a patient with a fever.
    - RiskNot: Negligible (1%). These symptoms are suggestive of bacterial tonsillitis, so the risk of illness continuing or worsening without antibiotics is higher than 1%.

2. If the level for Symptoms was ‘Sore and red throat, and swollen lymph nodes in the neck’ or ‘Productive cough and runny nose’ then the following attribute level was **not** permitted:

    - RiskNot: Likely (20%). These symptoms are likely to be a viral sore throat or a cold. NICE Clinical Guidance CG69 states: “*These conditions are largely self-limiting and complications are likely to be rare if antibiotics are withheld*”. Hence we avoided scenarios that presented the risk of relapse or progression with no antibiotics as ‘Likely’.

Reference:

NICE Clinical Guidance CG69. Respiratory tract infections (self-limiting): prescribing antibiotics (2008). https://www.nice.org.uk/guidance/cg69, accessed 5/12/19

SECTION 7. Modelling the continuous variables as categorical

The models assume that the time and risk attributes can be represented as continuous variables, with a linear relationship with the outcome on the log-odds scale. In an exploratory analysis, these attributes were modelled and plotted as categorical variables.

The plot for risk due to delaying treatment suggested a threshold effect, with similar coefficients for the lower two attributes and a greater effect for the highest level. Conversely, the plot for duration of treatment showed a plateau, with the coefficient increasing with the attribute level then levelling off. Alternative models with these attributes as dichotomised variables showed marginally improved model fit though the results were qualitatively very similar. However, the models exhibited collinearity due to the restructuring of the variables, and there was insufficient information to determine exactly where the cut-off should be. As a result the linear approximation was retained in the models.
